# Identifying patients with *NTRK* fusion cancer

**DOI:** 10.1093/annonc/mdz384

**Published:** 2019-11-18

**Authors:** J P Solomon, R Benayed, J F Hechtman, M Ladanyi

**Affiliations:** 1 Department of Pathology, Memorial Sloan Kettering Cancer Center, New York, USA; 2 Human Oncology and Pathogenesis Program, Memorial Sloan Kettering Cancer Center, New York, USA

**Keywords:** *NTRK* fusions, ancillary testing, next-generation sequencing, tyrosine kinase inhibitor

## Abstract

Due to the efficacy of tropomyosin receptor kinase (TRK) inhibitor therapy and the recent Food and Drug Administration approval of larotrectinib, it is now clinically important to accurately and efficiently identify patients with neurotrophic TRK (*NTRK*) fusion-driven cancer. These oncogenic fusions occur when the kinase domain of *NTRK1*, *NTRK2* or *NTRK3* fuse with any of a number of N-terminal partners. *NTRK* fusions are characteristic of a few rare types of cancer, such as secretory carcinoma of the breast or salivary gland and infantile fibrosarcoma, but they are also infrequently seen in some common cancers, such as melanoma, glioma and carcinomas of the thyroid, lung and colon. There are multiple methods for identifying *NTRK* fusions, including pan-TRK immunohistochemistry, fluorescence *in situ* hybridisation and sequencing methods, and the advantages and drawbacks of each are reviewed here. While testing algorithms will obviously depend on availability of various testing modalities and economic considerations for each individual laboratory, we propose triaging specimens based on histology and other molecular findings to most efficiently identify tumours harbouring these treatable oncogenic fusions.

**Key messages**

*NTRK* fusions are seen in a few rare cancer types and occur infrequently in some common cancers. Accurate identification of *NTRK* fusion-driven cancer is clinically important and may be achieved using multiple methods. We propose triaging specimens for *NTRK* fusion testing based on histology and other molecular findings to most efficiently identify patients with these treatable oncogenic fusions.

## Oncogenic *neurotrophic tropomyosin receptor kinase* fusions

The neurotrophic tropomyosin receptor kinases are a family of transmembrane tyrosine kinases that are important players in neural development. The three members of the family, TRKA (NTRK1), TRKB (NTRK2) and TRKC (NTRK3), are encoded by the *NTRK1*, *NTRK2* and *NTRK3* genes, respectively, and each consists of an extracellular ligand-binding domain, a transmembrane region and an intracellular kinase domain [1]. Normally, physiological activation of the receptor through ligand binding activates the kinase domain, leading to receptor homodimerisation, phosphorylation and activation of downstream signalling pathways [2]. Although highly homologous, each receptor has a preferred ligand: TRKA has the highest affinity for neurotrophin nerve growth factor, TRKB has the highest affinity for brain-derived neurotrophic factor and neurotrophin-4 and TRKC has the highest affinity for neurotrophin-3 [1−5]. A number of splice variants have been characterised, particularly involving *NTRK1*, and these variants have been observed both in normal tissues and in human cancers such as neuroblastoma and acute myeloid leukaemia where it is thought that they may play a role in tumourigenesis [1, 2]. While some studies have identified somatic point mutations or amplification in the *NTRK* genes, such alterations have so far not been shown to be a driver of oncogenesis [2]. 

Constitutive activation of the tropomyosin receptor kinase (TRK) receptors and subsequent downstream pathways can occur through chromosomal inversions, deletions or translocations that result in an in-frame fusion of the C-terminal tyrosine kinase domain of any of the *NTRK* genes with an N-terminal fusion partner. A multitude of 5′ fusion partners have been described, and in virtually all cases, the fusion eliminates the ligand binding site, resulting in ligand-independent dimerisation and phosphorylation [2]. The first TRK fusion protein was originally described in a colorectal adenocarcinoma cell line, but even at the time of discovery, it was recognised that involvement of this particular oncogene in such a fusion was an uncommon event in colon cancer [6]. It was later discovered that infantile fibrosarcoma was characterised by an *ETV6*-*NTRK3* fusion involving a translocation of chromosomes 12 and 15 [7, 8], and this same fusion was subsequently also reported in secretory carcinoma of the breast and salivary gland, which now defines these subsets of carcinomas [9, 10]. *NTRK* fusions have also been reported in a subset of carcinomas of the thyroid, especially in patients with a history of exposure to radiation [11], and they are also rarely found in many other tumours, including carcinomas of the lung [12] and colon [13], gliomas [14, 15], other sarcomas [16], inflammatory myofibroblastic tumours [17] and melanocytic tumours [18, 19].

In recent years, clinical trials have shifted away from site-of-origin and histological subtype-specific designs and more towards basket trials, which are designed to test therapies targeted towards specific molecular mechanisms [20], and trials targeting *NTRK* fusions have been particularly successful. In one such recent trial, larotrectinib showed remarkable and durable efficacy against locally advanced and metastatic solid tumours harbouring an *NTRK* fusion [21]. Entrectinib, active against *NTRK* fusions as well as fusions involving *ROS1* and *ALK*, has also shown great efficacy in recent clinical trials [22, 23]. The success of larotrectinib has resulted in its subsequent fast-track approval by the Food and Drug Administration (FDA),* and therefore standard of care will now require accurate identification of patients who could benefit from this practice-changing therapy.

## Methods for detection

### Immunohistochemistry

**Figure 1.**
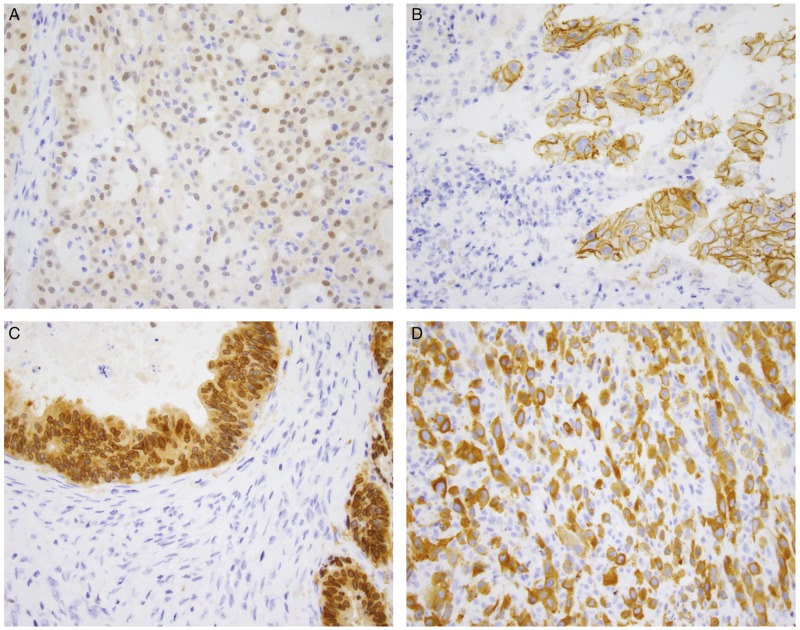
Patterns of immunohistochemical staining in *NTRK* fusion-positive tumours. (A) Secretory carcinoma of the salivary gland with an *ETV6-NTRK3* fusion shows weak to moderate nuclear and cytoplasmic staining. (B) Intrahepatic cholangiocarcinoma with a *PLEKHA6-NTRK1* fusion shows prominent membranous staining. (C) Gallbladder adenocarcinoma with an *LMNA-NTRK1* fusion shows strong cytoplasmic and perinuclear staining. (D) Metastatic thyroid carcinoma to soft tissue with a *TPM3-NTRK1* fusion shows strong cytoplasmic and membranous staining.

Immunohistochemistry to examine protein expression has several advantages. It is commonly used in clinical labs and is therefore relatively straightforward to implement and validate. It also has the benefits of being inexpensive, requiring only a single unstained slide and having a rapid turnaround time. The clone that is most often used and well-studied is clone EPR17341 (Abcam and Roche/Ventana), which reacts with a conserved proprietary peptide from the C-terminus of TRKA, TRKB and TRKC, and is therefore reactive with any of the oncogenic *NTRK* fusions. Positive staining has been defined as staining above background in at least 1% of tumour cells [24]. Initial studies have shown sensitivity ranging from 75% to 96.7% and specificity ranging from 92% to 100% [24−27]. However, the staining intensity has been shown to be variable, and staining pattern correlates with fusion partner (Figure 1) [25]. The fusion partner can direct the fusion protein to localise to other cellular compartments, in contrast to the membrane-associated expression of native TRK. One caveat is that recent studies have shown reduced sensitivity for *NTRK3* fusions [24]. In our experience, e.g. we have found that sensitivity for *NTRK1* and *NTRK2* fusions was 96% and 100%, respectively, while sensitivity for *NTRK3* fusions was 79% [28]. In addition, immunohistochemistry seems to have variable specificity according to tumour type. While the antibody appears to have 100% specificity in carcinomas of the colon, lung, thyroid, pancreas and biliary tract, decreased specificity is seen in breast and salivary gland carcinomas, as cytoplasmic staining can occasionally be seen. Specificity is lower in sarcomas, particularly those with neural or smooth muscle differentiation as wild-type TRK protein is physiologically expressed in neural and smooth muscle tissue [27, 28].

### Fluorescence *in situ* hybridisation

Fluorescence *in situ* hybridisation (FISH) can detect large structural variants at the DNA level and is often used in the clinical laboratory to detect oncogenic fusions in solid tumours. A commercial break-apart probe is available for the *ETV6* gene (Abbott, Chicago, IL) where separation of a green signal at the centromeric 3′ end of *ETV6* and an orange signal at the 5′ end of *ETV6* indicates a structural variant involving the gene. In cases that are histologically suggestive of *ETV6-NTRK3* fusions, such as infantile fibrosarcoma, congenital mesoblastic nephroma or secretory carcinoma of the salivary gland or breast, such testing can be useful to confirm the translocation [29]. Since fusions in other cancers can involve any of the *NTRK* genes and any of a number of partners through either balanced or unbalanced translocation or large deletions, only examining the *ETV6* gene would miss many oncogenic *NTRK* fusions. To this end, break-apart probes for the three *NTRK* genes have been used to identify fusions and are commercially available from multiple sources [16, 30–32]. Theoretically, break-apart probes have adequate sensitivity and specificity for chromosomal abnormalities, but there are practical technical considerations in the interpretation of break-apart FISH assays. In one study, short inversions and intrachromosomal translocations involving *ALK* resulted in a short split length using a break-apart probe. These short split lengths were difficult to distinguish from those seen in some normal cells, and therefore can result in false-negative results [33]. These findings would have particular relevance for *NTRK1* fusions, a majority of which are intrachromosomal events involving chromosome 1 [34]. For example, *LMNA-NTRK1* fusions are formed through an intrachromosomal deletion, which can result in a false-negative FISH due to insufficient splitting of the signals. In addition, while a positive result with a break-apart probe shows the presence of a structural variant involving the probed gene, whether the abnormality results in a functional transcribed fusion cannot be determined. Advantages of FISH include that the amount of material required is only a few unstained slides—usually one unstained slide per probe examined—and the turnaround time is usually only a few days.

### Reverse transcriptase polymerase chain reaction

Reverse transcriptase polymerase chain reaction (RT-PCR) can be used to detect the presence of transcribed RNA, and it can be used either qualitatively or quantitatively to detect the presence of a single oncogenic fusion for which both fusion partners are known. For *NTRK* fusions, however, because of the number of different fusion partners and breakpoints involved, the utility of RT-PCR for individual fusion transcripts is limited and has been used in the past mainly to detect canonical *ETV6-NTRK3* fusions. Even though this method may prove difficult to obtain direct evidence of a fusion, RT-PCR could be used to examine differences in expression of the 5′ versus the 3′ ends of a gene, as this has been shown to be associated with the presence of a translocation [35, 36]. For *NTRK*, which is not expressed in most normal tissues, the 3′ kinase domain would be transcribed at a much higher level than the 5′ extracellular domain in tumour tissue that harbours an *NTRK* fusion. Such an assay could thereby provide indirect evidence of an *NTRK* fusion.

### DNA-based next-generation sequencing

In DNA-based next-generation sequencing (NGS), tumour DNA is extracted from formalin-fixed paraffin-embedded (FFPE) tissue and then sequenced to investigate whether specific alterations are present in the tumour. Although there are a number of different library preparation and sequencing solutions available, there are two main approaches to isolating the genes of interest for sequencing. Amplicon-based methods use PCR primers to amplify the areas of interest, and while this method is suitable for detecting point mutations and small indels in a small panel of genes, the detection of gene fusions, which usually involve intronic breakpoints, is limited. Targeted hybridisation capture-based NGS assays on the other hand use capture probes that hybridise to the areas of interest in the genome. This non-biased approach enables deep sequencing of exons of key cancer-related genes for the detection of single point mutations, indels and copy number variations. In addition, the introns of specific genes known to be involved in functional gene fusions can be tiled with probes (baits) to detect these rearrangements [25, 37]. It should be noted, however, that some introns, such as those in *NTRK3*, are extremely long (spanning 193 KB) and would not be feasible to cover. If covered, those introns would constitute a large percentage of the panel size resulting in coverage reduction of other exonic regions and the overall assay sensitivity. Furthermore, some of these intronic regions cannot be effectively captured even if desired because they contain repetitive elements which cannot be tiled with unique capture probes and therefore cannot yield reads that can be reliably mapped back to that intron (poor mapping quality). Therefore, sensitivity of DNA-based NGS suffers if fusion breakpoints involve long intronic regions that cannot be covered by hybridisation-capture probes. For example, MSK-IMPACT, the DNA-based NGS assay used at Memorial Sloan Kettering Cancer Center, interrogates introns 3 and 7 through 12 in *NTRK1*, intron 15 in *NTRK2* and introns 4 and 5 in *ETV6*, the most common *NTRK3* fusion partner. However, because of the aforementioned issues involving coverage of the *NTRK3* introns, fusions involving *NTRK3* other than *ETV6*, are not covered by the assay [25, 28, 37]. These considerations are not limited to MSK-IMPACT, as other widely used cancer gene panels such as FoundationOne CDx also only assess these same intronic regions [38].

One drawback to DNA-based NGS is that when novel structural variants are detected, it can be difficult to determine whether such an event results in a functional expressed fusion. In these cases, ancillary testing with an orthogonal method, such as RNA-based NGS can be carried out. Other drawbacks include turnaround time, which is significantly longer than immunohistochemistry or FISH, and that more material is required for testing. On the other hand, a major advantage of DNA-based NGS testing is that many genomic events can be interrogated, allowing for simultaneous direct assessment of point mutations, indels, copy number variants and tumour mutation burden in addition to DNA-level gene fusions. Information gained from using this method includes MAPK driver status, which can be used to triage any follow-up testing (see Testing algorithm considerations section). Finally, this method is also effective for monitoring patients with *NTRK* fusions for development of resistance mutations. Recent studies have observed p.G667C and p.G595R mutations in *NTRK1* and p.G696A mutations in *NTRK3* that confer resistance to TRK inhibitor therapy [21, 39]. Using DNA-based NGS to monitor for tumour evolution is therefore useful in patients with *NTRK* fusion-positive cancers treated with TRK inhibitor therapy.

### RNA-based NGS

RNA-based sequencing presents several advantages over DNA. The introns are spliced out in the RNA, which removes the technical limitations of intronic coverage. In addition, detection of RNA-level fusions provides direct evidence that they are functionally transcribed, and analysis of the spliced sequence can determine whether the protein would be translated and in-frame. Fusion transcripts can also be detected with high confidence in the RNA of low tumour purity samples because gene fusions are often highly expressed in the tissue.

Detection of the fusion transcript by RNA-based NGS can be carried out using a few different enrichment methods. In all technologies, the RNA library is first converted to cDNA through reverse transcription. Then, for amplicon-based panels, PCR is used to amplify the sequences of interest. In assays that use standard multiplex PCR, both the driver gene and the fusion partner must be known and the two gene-specific PCR primers must be present in the assay for amplification to occur. In one study that used such a multiplexed amplicon approach using a panel of primer pairs for 169 gene fusions between 19 target genes and 94 fusion partners, sensitivity for fusion detection was determined to be 86% [40, 41]. However, when incorporating the read count information to determine 5′/3′ ratios, as described above for RT-PCR, sensitivity was increased to 100% [40].

A more sensitive and specific targeted amplicon-based method for fusion gene detection is anchored multiplex PCR that is commercially available through ArcherDX. In this technology, an Illumina sequencing adaptor is ligated to both ends of the cDNA. In the PCR steps, a gene-specific primer hybridises to the gene of interest, while a universal primer hybridises to the ligated adaptor sequence. Since fusion breakpoints are usually within introns, the gene-specific primers are often complementary to regions at the ends of exons such that the PCR products span exon boundaries. By using this method of sequencing, only one fusion partner needs to be targeted, and therefore novel fusion partners can be characterised [42, 43]. In our laboratory at Memorial Sloan Kettering, we have shown that this technology is able to sensitively and specifically identify fusion transcripts and alternative transcripts resulting from splicing alterations [43, 44].

Capture-based approaches for either targeted or whole transcriptome sequencing assays can also be used. After reverse transcription to convert extracted RNA to cDNA, hybridisation capture is carried out using capture probes in a method similar to that used in DNA-based sequencing. With this method, only one fusion partner needs to be known. The clinically validated assay, OSU-SpARKFuse, which uses such an approach, demonstrated 93.3% sensitivity and 100% specificity for fusion detection [45].

One of the main drawbacks to working with RNA is its lability. RNA can be extracted from FFPE tissue, but because it is susceptible to fragmentation and degradation, especially in older material, adequate quality control is required. Methods to interrogate RNA quality can be analysis of RNA fragment size distribution and examining amplification of a housekeeping gene in a quantitative PCR-based assay [46]. For assessing quality of the sequencing assay, one can determine the ratio of RNA reads to DNA reads and examine sequencing coverage and depth for the RNA reads.

Finally, it should be noted that some commercially available platforms are able to simultaneously assess both RNA and DNA. The DNA and RNA libraries are prepared separately, but then can be combined for analysis in a single sequencing run. The Oncomine Comprehensive Assay by ThermoFisher, which uses amplicon-based technology, covers 161 cancer-related genes and can detect fusions involving all three *NTRK* genes. Per ThermoFisher’s technical specifications, the assay can be carried out with as few as three FFPE slides or as little as 10 ng of DNA or RNA. The TruSight Oncology 500 assay by Illumina uses hybridisation-capture technology to interrogate DNA-level alterations involving 523 cancer-related genes, and it also sequences RNA-transcripts to detect fusions involving any of 55 genes, including all three *NTRK* genes.

## Testing algorithm considerations

**Figure 2.**
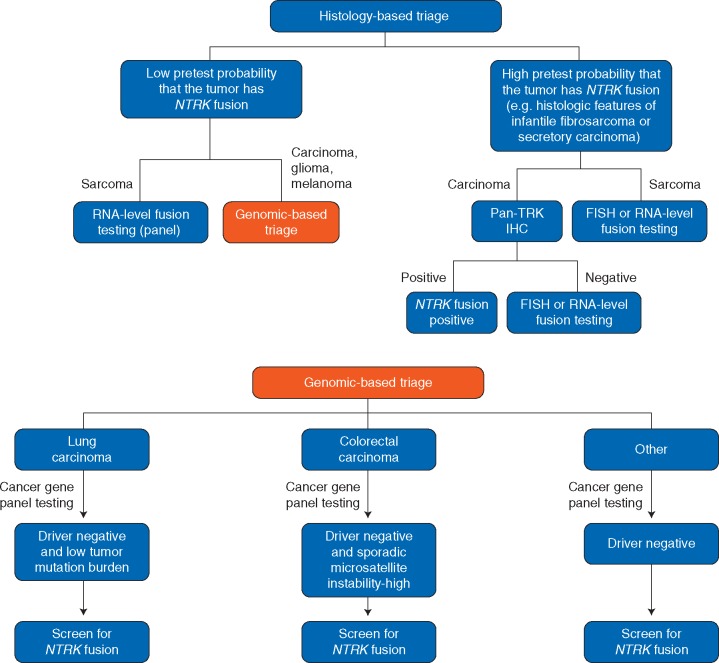
Diagnostic algorithm for *NTRK* testing. Histology-based triaging should first be carried out to separate the rare cancer subtypes that commonly have *NTRK* fusions from those that have a low pre-test probability of *NTRK* fusions. In the tumours that often have oncogenic *NTRK* fusions, confirmatory methods can be used. In secretory carcinomas, pan-TRK immunohistochemistry can be used as an initial screen, but if negative, additional testing with FISH or RNA-level fusion testing should be used. In sarcomas, immunohistochemistry should be eschewed due to its lower specificity. It is also worth noting that comprehensive fusion testing (for all major sarcoma fusions) is increasingly being carried out as a first-line test in sarcomas rather than waiting for results from a DNA-based triage as one would in carcinoma. We therefore recommend inclusion of *NTRK* primers in comprehensive sarcoma fusion test panels. In cancers with a low pre-test probability of *NTRK* fusion, such as most carcinomas, gliomas and melanomas, molecular testing such as DNA-based cancer gene panels is often carried out, and driver status can therefore be used to narrow down the tumours that should undergo further screening for oncogenic fusions, as *NTRK* fusions are typically mutually exclusive with other common mitogenic driver alterations that activate MAPK signalling. The resulting ‘driver-negative’ cases are therefore likely enriched for *NTRK* fusions and these can be screened for by IHC or an RNA-based fusion panel assay. For lung and colorectal cancer, we highlight how to further enrich for *NTRK* fusions in settings where broad, routine screening is not possible.

While the importance of identifying patients that could benefit from targeted therapy cannot be understated, feasibility and economic considerations should also be taken into account when creating testing algorithms and guidelines. Triaging specimens should be based not only on tumour type and its pre-test probability for *NTRK* fusions, but also on the availability of clinically validated methodologies along with their positive and negative predictive values (Figure 2). Overall, a comprehensive diagnostic algorithm that is appropriate for all clinical situations and laboratories is difficult to propose and depends on all of these factors. Here, we discuss a few considerations based on the observation that a few rare cancer types commonly harbour *NTRK* fusions whereas common cancer types rarely harbour *NTRK* fusions. However, in absolute numbers, the latter group contributes the majority of patients with *NTRK* fusions.

### Histology-based triaging

The rare cancer subtypes that commonly harbour *NTRK* fusions include secretory carcinomas of the breast and salivary glands, infantile fibrosarcoma, congenital mesoblastic nephroma and paediatric papillary thyroid cancer. For these tumours, a histology-based testing algorithm is preferred and confirmation of the *NTRK* fusion should be carried out using a preferred method that has high specificity, such as FISH or DNA-based NGS. If negative by a single method, additional testing should be carried out.

### Mass screening-based detection

The second group includes many cancer types with a low probability (<1%) of harbouring *NTRK* fusions. These low probability cancers include lung cancer, breast cancer, colorectal cancer, pancreatic cancer, cholangiocarcinomas, adult papillary thyroid cancer (2%), melanomas, gliomas and sarcomas (gastrointestinal stromal tumour, uterine and soft tissue). Mass screening approaches include comprehensive molecular evaluation using broad NGS that includes assessment for *NTRK* fusions as part of broad panels, as described above, or routine screening by immunohistochemistry, with the caveats and limitations stated above. Mass screening-based detection is not algorithmic and involves no triaging; it refers to testing all patients either by immunohistochemistry or by comprehensive DNA- and RNA-based panels. Therefore, it is intrinsically inefficient if one is solely screening for an alteration with very low prevalence, such as *NTRK* fusions.

### Genomic-based triaging

In many cases in the group of cancer types with a low probability (<1%) of harbouring *NTRK* fusions, one can triage tumours that are more likely to harbour *NTRK* fusions based on their genomic profiles. *NTRK* fusions are typically mutually exclusive with *KRAS*, *NRAS*, *BRAF*, *MAP2K1*, *EGFR*, *ALK*, *RET*, *ROS1*, *KIT*, *PDGFRA* and other common mitogenic ‘driver’ alterations that activate MAPK signalling. Thus, excluding cases in which a known mitogenic driver has been identified (‘driver-negative’) can significantly narrow down the number of cases to be systematically screened for *NTRK* fusions. For example, in a recent study, we observed a high yield of RNA sequencing for targetable kinase fusions, including *NTRK* fusions, in driver-negative lung adenocarcinomas that also showed low tumour mutation burden [44]. In another similar study, Cocco et al. examined a cohort of 2314 colorectal adenocarcinomas with MSK-IMPACT, identifying 21 cases with kinase fusions, including 8 with *NTRK* fusions. Of the 21 cases with kinase fusions, a majority (57%, 12/21) were present in cases that had microsatellite instability. Of a cohort of 24 colorectal carcinoma cases that exhibited microsatellite instability due to *MLH1* promoter methylation and that did not have driver mutations in *RAS* or *BRAF*, 10 harboured kinase fusions, including 6 that had *NTRK* fusions. Overall, the findings from this study identify a subset of colorectal carcinomas for which there should be a high suspicion of kinase fusions [47]. Therefore, it may be most efficient to focus ancillary testing on colonic adenocarcinomas that have microsatellite instability and that lack other conventional driver mutations. For lung and colorectal cancer, such approaches highlight how to further enrich for *NTRK* fusions in settings where broad, routine screening is not possible. We do not propose restricting further testing to these subsets of driver-negative lung or colorectal cases but mainly wish to emphasise which types of cases should be of highest priority for further screening.

## Discussion

### Conclusions

Oncogenic *NTRK* gene fusions occur in many different tumour types. While present in a majority of certain rare tumours, they are also rarely present in many common cancers. With the recent FDA approval of *NTRK* targeted therapy and the marked and durable responses these agents produce in patients with *NTRK* fusion-positive cancers, identification of tumours harbouring these fusions has become essential. Our approach echoes the recently published recommendations of the ESMO Translational Research and Precision Medicine Working Group [48], but we place more emphasis on genomic-based triage. Further downstream in the cancer care timeline, as we more routinely detect *NTRK* fusions that make patients eligible for TRK inhibitors, we will also have to consider how our molecular diagnostic tests detect mechanisms of acquired resistance in these patients, both on-target second site mutations in the *NTRK* kinase domain [49, 50] and alterations that activate bypass signalling pathways [51].

## Footnotes

* Note added in proof: The European Medicines Agency granted marketing authorisation for larotrectinib on 23 September 2019 as monotherapy for the treatment of adult and paediatric patients with solid tumours that display a neurotrophic tyrosine receptor kinase (*NTRK*) gene fusion, and who have disease that is locally advanced, metastatic or where surgical resection is likely to result in severe morbidity, and who have no satisfactory treatment options.
